# Veno-arterial extracorporeal membrane oxygenation for severe fever with thrombocytopenia syndrome with fulminant myocarditis: a case report

**DOI:** 10.1186/s12879-021-06446-4

**Published:** 2021-08-11

**Authors:** Uh. Jin Kim, Hyukjin Park, Kye Hun Kim, Dong Min Kim, Seung Eun Kim, Seung Ji Kang, Kyung-Hwa Park, Sook In Jung

**Affiliations:** 1 Department of Infectious Diseases, Chonnam National University Medical School, 42 Jaebongro, Dong-gu, Gwangju, 61469 South Korea; 2 Department of Cardiology, Chonnam National University Hospital, Gwangju, Republic of Korea; 3 Division of infectious Diseases, Department of Internal Medicine, Chosun University College of Medicine, Gwangju, Republic of Korea

**Keywords:** SFTS, Myocarditis, ECMO, Case report

## Abstract

**Background:**

The clinical spectrum of severe fever with thrombocytopenia syndrome (SFTS) is wide, which can range from fever to multiple organ failure. Conservative therapy plays a key role in the treatment of SFTS. However, severe cases of SFTS, such as fulminant myocarditis, may require mechanical hemodynamic support.

**Case presentation:**

This report presents a case of a 59-year old woman diagnosed with SFTS by reverse-transcription polymerase chain reaction. The patient had no initial symptoms of cardiac involvement and rapidly developed hemodynamic instability 3 days after hospitalization. She suffered from chest pain and had elevated cardiac enzymes. In the absence of atrio-ventricular conduction abnormalities, left ventricular dysfunction, and coronary artery abnormalities by coronary angiography, she was diagnosed with fulminant myocarditis. At that time, her pulse rate nearly dropped to 0 bpm and she developed near complete akinesia of the heart despite vasopressor administration. Veno-arterial extracorporeal membrane oxygenation (ECMO) was initiated with other supportive measures and she fully recovered after 21 days.

**Conclusions:**

This case indicates that SFTS can cause fulminant myocarditis even without evidence of cardiac involvement at presentation. When symptoms and/or signs of acute heart failure develop in patients with SFTS, myocarditis should be suspected and the patient should be promptly evaluated. Additionally, mechanical hemodynamic support like ECMO can be a lifesaving tool in the treatment of fulminant myocarditis.

**Supplementary Information:**

The online version contains supplementary material available at 10.1186/s12879-021-06446-4.

## Background

Severe fever with thrombocytopenia syndrome (SFTS) was first discovered in China in 2007 and is now a worldwide problem. SFTS has been detected in other Asian regions including Japan and Korea as well as the Mediterranean and the United States [1].

The most common clinical manifestations of SFTS are fever, vomiting, diarrhea, thrombocytopenia, and leukopenia. However, severe cases include multiple organ failure, disseminated intravascular coagulopathy, and neurologic manifestations. Myocarditis caused by viral SFTS is a rare complication with a prevalence of 4.2% in Korea [2]. Nonetheless, myocarditis is a critical complication that can quickly lead to death without proper management.

To manage fulminant myocarditis, prompt diagnosis and supportive measures such as ventricular assist devices or extracorporeal membrane oxygenation (ECMO) may be required until recovery of cardiac function [3]. This case report describes a case of fulminant myocarditis caused by SFTS, in which the patient successfully recovered after veno-arterial (VA) ECMO and immunosuppressive therapy (IST).

## Case presentation

A 59-year-old woman who lives in a rural area of Republic of Korea presented to the emergency center of a tertiary hospital with a fever that started 3 days prior. She had no known underlying diseases. On admission, she had blood pressure of 90/40 mmHg, pulse rate of 68 beats per minute (bpm), respiratory rate of 20 breaths per min, and a body temperature of 38.3 °C. She had no memory of any insect bites, but reported that she had rabbits, dogs, and chickens living on her property. Neither eschar nor skin rash were observed on physical examination.

**Fig. 1:**
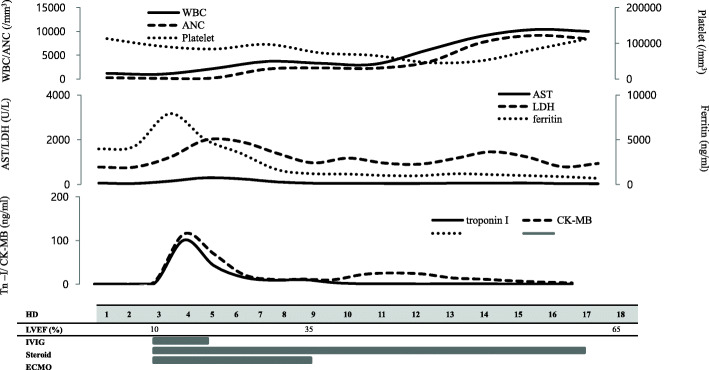
Clinical course of the patient during admission

**Fig. 2:**
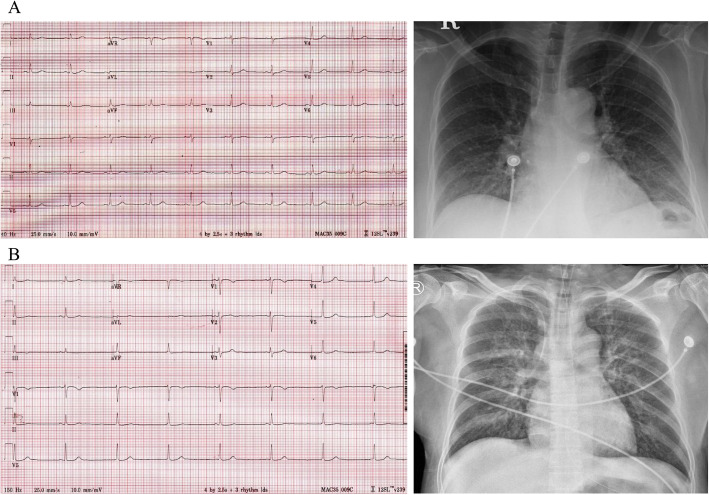
Electrocardiography (ECG) at presentation. ECG showing a normal sinus rhythm on day 1. A chest X-ray on day 1 is also shown (panel **A**). ECG showing the development of fulminant myocarditis with sinus bradycardia, temporary sinus arrest, and junctional escape beats on day 3. A chest X-ray from day 3 is also shown (panel **B**)

After a series of tests, laboratory tests revealed bicytopenia with a white blood cell count of 1200/mm^3^ (neutrophil count 280/μL), hemoglobin level of 13.9 g/dL, and a platelet count of 113,000/mm^3^. The serum levels of C-reactive protein, ferritin, aspartate aminotransferase (AST), alanine transaminase (ALT), and lactate dehydrogenase (LDH) were 0.05 mg/dL, 3970.62 ng/mL, 67 U/L, 28 U/L, and 781 U/L, respectively. Serum cardiac enzymes including troponin I and creatine kinase-muscle/brain (CK-MB) isoenzyme were all within normal range. The clinical course of the patient’s laboratory findings is demonstrated in Fig. 1. A baseline electrocardiography (ECG) displayed a normal sinus rhythm and there was no definite cardiomegaly nor pulmonary congestion in the chest X-ray (Fig. 2, panel A).

Based on the clinical manifestations, history of raising livestock in rural area, and laboratory finding, tick borne disorder such as SFTS was suspected. In addition, hematologic disorder cannot be ruled out. To determine a microbiological cause of fever, blood cultures were ordered. Reverse-transcription polymerase chain reaction (RT-PCR) was executed to screen for the SFTSV medium (M) segment in blood, indirect immunofluorescence was performed to screen for scrub typhus and Hantaan virus, and passive hemagglutination testing was performed to screen for *Leptospira*. A bone marrow sample was also taken to evaluate the grade of bicytopenia. The sample revealed decreased trilineage cells with hypocellular marrow, which suggested marrow failure due to systemic infection.

On hospital day (HD) 3, the patient complained of chest discomfort. Subsequently, her blood pressure dropped to 55/37 mmHg and she developed bradycardia (43 bpm). The patient’s heart rhythm also switched to an alternating rhythm of sinus bradycardia and junctional bradycardia. A chest X-ray displayed distinct pulmonary edema (Fig. 2, panel B). At that time, the serum troponin I titer was elevated (1.60 ng/mL).

Laboratory examinations from HD 3 again revealed bicytopenia with a white blood cell count of 2200/mm^3^ (neutrophil count 230/μL), hemoglobin level of 12.7 g/dL, and platelet count of 84,000/mm^3^. The serum levels of C-reactive protein, ferritin, and LDH were 0.01 mg/dL, 7921.17 ng/mL, and 1222 U/L, respectively. Bedside echocardiography revealed severe left ventricular (LV) systolic dysfunction (LV ejection fraction 10%) with normal LV chamber size and a mildly edematous LV posterior wall. An additional cardiac echography file shows this in more detail (see Additional file 1).

The patient then suddenly developed an atrio-ventricular conduction abnormality simultaneously with LV dysfunction. As such, it was suspected that the patient was experiencing a myocardial infarction of the right or left circumflex coronary artery, or fulminant myocarditis involving the conduction system. A coronary angiography was performed immediately, which revealed good distal flow through both coronary arteries and a lack of significant stenosis. Due to the patient’s severe orthopnea, agitation, and refractory shock, an endomyocardial biopsy (EMB) could not be performed.

Despite intravenous administration of norepinephrine and dopamine, the patient’s blood pressure did not recover. Therefore, VA ECMO was applied to the patient via the femoral vein and artery. The patient’s blood pressure dropped to nearly 0 mmHg and she developed a near total akinesia of the heart (Additional file 2) in the time it took to initiate ECMO, but cardiopulmonary resuscitation was not performed.

On the same day 3, the patient’s blood test result came back positive for SFTSV. Consequently, immune suppressive therapy (IST) with prednisolone (1 mg/kg/day) and 3 days of intravenous immunoglobulin (20 g/day) was initiated. In addition, the patient’s plasma was tested for a panel of 12 different cytokines. Among the panel, the concentrations of interleukin (IL)-6, IL-10, and tumor necrosis factor (TNF)-α were 3.52 pg/mL, 2.56 pg/mL, and 20.89 pg/mL, respectively. These levels were much higher than the mean values of ten Korean healthy adults, measured in our lab for research purposes, which were 1.55 pg/mL, 1.40 pg/mL, and 3.25 pg/mL, respectively. Levels of platelet derived growth factor (PDGF)-BB were also lower (347.94 pg/mL) compared to healthy adults (2347.76 pg/mL).

On HD 4, the patient still had bicytopenia with a white blood cell count of 2700/mm^3^ (neutrophil count 1760/μL), hemoglobin level of 11.0 g/dL, and a platelet count of 75,000/mm^3^. The serum levels of C-reactive protein, AST, ALT, and LDH were 0.01 mg/dL, 300 U/L, 68 U/L, and 1709 U/L, respectively. The patient’s troponin level was 101.56 ng/ml and the level of mass CK-MB was 115.4 ng/mL.

On HD 7, the patient’s heart started to beat again (Additional file 3). Further, on HD 12, she was successfully weaned from VA ECMO. By HD 21, cardiac function completely recovered (Additional file 4) and the patient was discharged without significant complications.

## Discussion and conclusions

To the best of our knowledge, this is the first case of successful resuscitation and recovery of cardiac arrest using ECMO and IST following an episode of fulminant myocarditis caused by SFTS. This case provides clinically important insight in the management of SFTS.

First, since SFTS can only be diagnosed if a clinician suspects it, in an area where SFTS is endemic, clinicians should always be aware of SFTS in patients with atypical manifestations of fever and cytopenia. A thorough history should also be taken to identify SFTS risk factors. Second, an early suspicion of myocarditis is critical for managing SFTS in patients with shock. This is because it could quickly evolve into cardiac arrest, even in patients without any evidence of myocardial involvement at the time of presentation. Third, prompt echocardiographic examination is essential in such circumstances and circulatory support like ECMO should be initiated immediately.

There are various pathogens that can cause acute heart failure (HF) during infection. In acute HF, where an ischemic insult has been excluded, immune and inflammatory mediated myocardial injury (such as viral myocarditis) should be suspected in a setting of viral disease. Immediate echocardiography is essential to evaluate cardiac function and structure. A distinct LV systolic dysfunction without LV dilation usually implies acute HF. In such circumstances, acute coronary syndrome should be ruled out and other possible causes quickly identified.

Although the diagnostic gold standard for managing myocarditis is EMB, it is infrequently used because of the lack of available facilities, clinical experience, and clinical instability [4]. In this case, an EMB was not possible given the patient’s hemodynamic instability and the SFTS diagnosis at the time of HF, which indicated viral myocarditis. Even without available EMB results, the clinical presentation of the patient fulfilled the diagnostic criteria of myocarditis (ECG/troponin/echocardiography) in the absence of significant coronary arterial disease, known pre-existing cardiovascular disease, or extra-cardiac causes that could explain the syndrome [3].

Recent studies have shown that direct viral invasion of cardiomyocytes and secondary immune reactions are vital for the development of viral myocarditis [5]. Immunohistochemistry of a severe SFTS patient revealed that SFTSV can directly infect multiple organs, including the heart [6]. In addition, cytokines are considered to play a key role in the pathogenesis of SFTS. Further, in severe SFTS cases that include heart failure, cytokines such as IL-6, IL-10, TNF-α, and interferon-γ have been reported to be elevated compared to healthy adults [7, 8]. Similar to previous reports, serum cytokines including IL-6, IL-10, and TNF-α were higher compared to healthy adults in this case, which explains the possible pathogenesis of viral myocarditis by SFTSV.

Viral myocarditis has a variable clinical presentation. It ranges from mild chest pain to fulminant myocarditis with rapid progression within hours, or from a severe febrile respiratory syndrome to cardiogenic shock, which can progress into multiple organ failure, as in this case. In viral myocarditis, myocardial damage and edema can occur as early as week 1 in 95% of patients [9]. Myocardial damage and edema lead to arrhythmia and fibrosis with an increased risk of sudden cardiac death. Among these risks, severely reduced LV ejection fraction and life-threatening arrhythmias are considered high risk for sudden cardiac death in myocarditis [10]. Therefore, early diagnosis and close monitoring is crucial in the management of acute viral myocarditis. This is especially important because SFTS is usually managed by an infectious disease specialist rather than cardiologists or cardiothoracic surgeons. Clinicians should not rule out the possibility of fulminant myocarditis in SFTS patients with refractory shock.

The usefulness of ECMO in patients with acute or fulminant viral myocarditis with hemodynamic instability has been reported in infections caused by enterovirus and Coxsackie virus [11, 12]. However, there are no reports showing that ECMO support is successful in treating myocarditis secondary to SFTS.

Following diagnosis of acute HF, it is important to first manage the cause of HF to prevent further myocardial damage. However, no specific treatment has been established for most viral myocarditis cases. Even in cases of acute HF that have been appropriately managed to stop further myocardial damage, the recovery of myocardial function itself takes time. In this case, the patient’s heart completely lost myocardial function within 24 h of the first sign of myocarditis. Since it took 9 days for her heart to efficiently circulate blood on its own, the ECMO support was essential for recovery.

ECMO is a useful tool for hemodynamic support in various clinical situations. These include acute respiratory distress syndrome and refractory cardiogenic shock [4]. ECMO is expected to be a life-saving tool in such cases of refractory shock as a bridge therapy until definite therapies show their effect. Early communication with a cardiology specialist about mechanical support such as ECMO may be the lifesaving step for fulminant myocarditis caused by SFTS with hemodynamic instability.

In conclusion, a case of fulminant myocarditis caused by SFTSV was described, which was successfully managed by veno-arterial ECMO and IST. In patients with SFTS, myocarditis should be suspected and diagnosed with immediate echocardiography upon the development of symptoms and/or signs of acute HF, even if there is no evidence of cardiac involvement at presentation. When the diagnosis of myocarditis is established, proper medical treatment should be administered without delay. In cases of fulminant myocarditis with hemodynamic instability, ECMO can be a good therapeutic option as a bridge until myocardial recovery.

## Supplementary Information

**Additional file 1.** Echocardiography at the time of fulminant myocarditis diagnosis on hospital day 3 (HD 3). The left ventricle (LV) is mildly edematous with normal LV size and a marked systolic dysfunction.

**Additional file 2.** Echocardiography at the time of ECMO initiation on HD 3. The LV became nearly akinetic only several hours after the initial echocardiography.

**Additional file 3.** Echocardiography after 4 days of immunosuppressive therapy (IST) on hospital day 6 (HD 6). Myocardial function of basal lateral and posterior segments of the LV and the right ventricle was recovered.

**Additional file 4.** Echocardiography after 18 days of IST on HD 20. Cardiac function and structure were completely recovered.

## Data Availability

Data sharing is not applicable to this article as no datasets were generated or analysed during the current study.

## Abbreviations

SFTS: Sever fever with thrombocytopenia syndrome
ECMO: Extracorporeal membrane oxygenation
VA: Veno-arterial
IST: Immunosuppressive therapy
BPM: beats per minute
AST: Aspartate aminotransferase
ALT: Alanine transaminase
LDH: Lactate dehydrogenase
CK-MB: Creatine kinase-muscle/brain
ECG: Electrocardiography
RT-PCR: Reverse-transcription polymerase chain reaction
M: Medium
HD: Hospital day
LV: Left ventricular
EMB: Endomyocardial biopsy
IL: Interleukin
TNF: Tumor necrosis factor
PDGF: Platelet derived growth factor
HF: Heart failure
